# The Use of Twitter by Trauma and Orthopaedic Surgery Journals: Twitter Activity, Impact Factor, and Alternative Metrics

**DOI:** 10.7759/cureus.1931

**Published:** 2017-12-10

**Authors:** Hannah Hughes, Andrew Hughes, Colin G Murphy

**Affiliations:** 1 Department of Trauma and Orthopaedic Surgery, St. Vincent's University Hospital, Dublin, Ireland; 2 Department of Trauma and Orthopaedic Surgery, Galway University Hospital; 3 Department of Trauma and Orthopaedic Surgery, Galway University Hospital , Galway, IRL

**Keywords:** twitter, social media, klout score, altmetric, alternative metrics, impact factor, orthopaedic journals, trauma and orthopaedics, trauma and orthopaedics, influence

## Abstract

Aim

Social media (SoMe) platforms have become leading methods of communication and dissemination of scientific information in the medical community. They allow for immediate discussion and widespread engagement around important topics. It has been hypothesized that the activity on Twitter positively correlates with highly cited articles. The purpose of this study was to analyze the prevalence and activity of Trauma and Orthopaedic Surgery journals on Twitter, with the hypothesis that impact factor is positively associated with Twitter usage.

Methods

The top 50 Trauma and Orthopaedic Surgery journals, ranked by 2016 Impact Factor were analyzed. The Twitter profiles of each journal or affiliated society were identified. Other SoMe platforms used were also recorded. Twitonomy software (Digonomy Pty Ltd, New South Wales, Australia) was used to analyze the Twitter profiles over a one-year period. Twitter Klout Scores were recorded for each journal to approximate the SoMe influence. Altmetric Scores (the total number of mentions via alternative metrics) were also recorded. Statistical analysis was carried out to identify correlations between journal Impact Factors, SoMe activity, Twitter Klout Scores and Altmetric Scores.

Results

Twenty-two journals (44%) had dedicated Twitter profiles. Fourteen journals (28%) were associated with societies that had profiles and 14 journals (28%) had no Twitter presence. The mean Impact Factor overall was 2.16 +/- 0.14 (range, 1.07-5.16). The journals with dedicated Twitter profiles had higher Impact Factors than those without (mean 2.41 vs. 1.61; P=0.005). A greater number of Twitter followers were associated with higher Impact Factors (R2 0.317, P=0.03). Journals with higher Twitter Klout Scores had higher Impact Factors (R2 0.357, P=0.016). Altmetric Score was positively associated with Impact Factor (R2 0.310, P=0.015). Journals with higher numbers of retweets (virtual citations in the Twittersphere) had higher Altmetric Scores (R2 0.463, P=0.015).

Conclusion

Trauma and Orthopaedic Surgery journals with dedicated Twitter profiles have higher Impact Factors than those without. Altmetrics is likely to play a significant role in the literature evaluation going forward along with the traditional metrics. The engagement with Twitter by Trauma and Orthopaedic surgeons should be encouraged.

## Introduction

Social media (SoMe) is a network of web-based and technological tools that aid the creation, sharing, and dissemination of information and ideas between individuals and communities online [1]. The use of social media has grown exponentially over the past decade, with a nearly 10-fold increase in its use in the United States alone [2]. Social media use in the field of medicine has grown rapidly in recent times. Twitter, in particular, has become a leading method of communication and dissemination of scientific information in the medical community, allowing for immediate discussion after seminal updates and widespread engagement around important topics. A notable increase in Twitter use has been observed at medical conferences globally, demonstrating its use as a platform for the promotion of engagement and collaboration amongst medical professionals [3-4].

Twitter is one of the world’s largest social media platforms. Founded 11 years ago, it is a microblogging site that provides information, opinions, and commentary about “what is happening in the world and what people are talking about right now” [5]. Twitter has over 320 million active monthly users who send approximately 6,000 tweets per second, totaling up to 500 million tweets per day [6]. Each user has a profile with an associated username beginning with the symbol ‘@’, e.g. @username. Twitter’s content is generated by these users, who share publicly available statements called ‘tweets’ from their profiles. Each tweet consists of a maximum of 140 characters and can contain text, images, videos, and hyperlinks. The use of ‘@username’ will mention the specific user in a tweet, alert them to a message or link others to @username’s profile. Users can also subscribe to other user’s tweets, known as ‘following’, and become ‘followers’. The content generated by these connections creates a ‘feed’ on a user’s profile, which can be viewed by others. Subsequently, a user’s tweets can be forwarded by other users to their own profile, also known as ‘retweeting’. Words or phrases can also be preceded by a hashtag (#). This categorizes tweets in a fashion that allows users to search for and interact with comments containing a specific word or phrase.

It has been hypothesized that activity on Twitter positively correlates with highly cited articles [7]. Whether it is the influence of SoMe or the quality of the article that predominantly influences its citation count is difficult to ascertain when traditional metrics, such as a journal’s Impact Factor, are looked at alone. This has necessitated the development of alternative metrics, also known as altmetrics. While a single research output may be present in one location online, the activity surrounding it may spread across a multitude of SoMe and online platforms [8]. Altmetrics evaluate the impact of these scholarly materials via online metrics, with an emphasis on data arising from social media outlets, including clicks, views, shares, downloads, saves, tweets, retweets, tags, mentions, bookmarks, commentary, as well as citations on Wikipedia [8-9]. This provides a complimentary online dimension to traditional citation metrics, i.e. those based on the number of times an article is cited in a database or publication.

Despite this, the data are still not available to determine if and how social media is used by Trauma and Orthopaedic Surgery journals and whether or not its use influences the Impact Factors of these journals. The purpose of this study is to analyze the use of Twitter by the leading Trauma and Orthopaedic Surgery journals, with the hypothesis that a journal’s Impact Factor is positively associated with a journal’s use of Twitter.

## Materials and methods

A search was conducted of InCites Journal Citation Reports 2016 (Thomson Reuters) on June 26, 2017, using “orthopedics and trauma” as the journal category filter. Non-orthopaedic, non-trauma related and non-English-language journals were excluded from the study. The remaining top 50 journals were ranked by Impact Factor and subsequently analyzed. The presence of each journal on Twitter was identified as having a dedicated journal specific Twitter profile, having an affiliated society Twitter profile or having no Twitter presence. Using InCites Journal Citation Reports, the year that each journal joined Twitter was recorded, as well as the journal’s Impact Factor that same year. The mean Impact Factor overall and of each category of journal Twitter presence was calculated. Whether or not journals experienced an increase in Impact Factor since joining Twitter was investigated. The use of other social media platforms was also recorded (Eg: Facebook, Instagram, etc.).

The Twitter profile of each journal was individually analyzed using Twitonomy software (Digonomy Pty Ltd, New South Wales, Australia) www. twitonomy.com on June 26, 2017. Twitonomy is an online application that links with Twitter to collate and process data related to user profiles. It has been used previously in Urology and Vascular Surgery research [10-11]. The parameters available for analysis include, but are not limited to, a user’s total number of tweets (140 characters maximum), retweets (forwarding another user’s tweet to one’s own profile), mentions (the use of @username in a tweet to directly reference said user), replies to tweets, hashtags, and followers (those who subscribe to another user’s profile), all of which can be exported and downloaded for further analysis.

Each Trauma and Orthopaedic journal Twitter profile was analyzed over a one-year period using Twitonomy software, from January 2016 to December 2016. The data collated included the total number of tweets, total number of retweets, number of followers, number of times the journal username is mentioned, replies to followers, as well as the year the journal created its Twitter profile. For journals with dedicated Twitter profiles, the change in Impact Factor between 2015 and 2016 was recorded. Clinical Biomechanics (@jclinbiomech) was excluded from this analysis as it joined Twitter in 2016 and thus had no measurable change in the Impact Factor.

To further assess SoMe influence, each journal’s Klout Score (www.klout.com) was measured via their Twitter profile on 16 June 2017. A Klout Score is generated by aggregating a large set of online reactions centered around the activity stimulated by a user or profile, such as clicks, likes, comments, reshares and purchase behavior [12]. It is a metric that can be used to measure a user’s influence across various online SoMe platforms and has been used in other medical research [11]. The Score ranges from one to 100. A higher Klout Score corresponds with greater influence. Whether a correlation exists between the age of a Twitter profile and its Klout Score was also investigated.

Another parameter of SoMe influence investigated was that of alternative metrics via the online analysis software Altmetric (www.altmetric.com). Altmetric collates data from multiple sources; bookmarks via reference managers like Mendeley; mentions, clicks and views on social media networks like Facebook and Twitter; citations via online information sites, such as Wikipedia. Each of the top 50 Trauma and Orthopaedic journals was searched on Altmetric and the datasets were downloaded as spreadsheets. The Altmetric Score (the total number of Altmetric mentions) was recorded for each journal. The presence of an association between the length of time a journal had been active on Twitter and the total number of mentions the journal had accumulated across the online and SoMe stratosphere was investigated.

Statistical analysis was conducted using Student’s t-test, Fisher contingency tables, and Pearson correlations. All statistical analysis was performed using Statistical Package for the Social Sciences (SPSS) version 24 (IBM Corp., Armonk, New York).

## Results

Overall, 72% of the journals were represented on Twitter, either by a dedicated Twitter profile or Twitter profiles of societies affiliated with the journals. Twenty-two journals (44%) had dedicated Twitter profiles. Of the remaining 28 journals with no dedicated Twitter profiles, 14 (28%) were associated with affiliated societies that had Twitter profiles, and 14 (28%) journals did not have a presence on Twitter. Of the journals with dedicated Twitter profiles, 21 out of 22 (95.5%) provided direct links to the journal website on their Twitter accounts.

Nineteen of the 22 (86.4%) journals with dedicated Twitter profiles had profiles on Facebook, 10 (45.5%) had profiles on Linkedin and six (27.3%) had Youtube profiles. As for the 14 journals with no presence on Twitter, two (14.3%) had a SoMe profile other than Twitter. The journals with a dedicated Twitter account were more likely to have a presence on other social media platforms, compared to journals with no Twitter account (p <0.001).

Association between Impact Factor, Twitter, and Twitter profile activity

The mean Impact Factor of all journals was 2.35 +/- 0.15 (range, 0.73-5.67). The Impact Factors of journals with dedicated Twitter profiles were higher than those without (mean 2.66 vs. 1.76; P=0.007). No statistically significant difference was found between the Impact Factors of the journals with affiliated society Twitter profiles and no Twitter presence (P=0.07).

Since the time of joining Twitter, 16 out of the 21 journals (76%) with dedicated Twitter profiles experienced an increase in the Impact Factor. Clinical Biomechanics (@jclinbiomech) was excluded from this analysis, as it joined Twitter in 2016 and thus had no measurable change in Impact Factor. There was no correlation between the number of years a journal had been active on Twitter and its Impact Factor. The mean change in the Impact Factor since joining Twitter was an increase of 0.35 +/- 0.09 (range, -0.30-1.41). The mean change in Impact Factor from 2015 to 2016 across all journals was an increase of 0.17 +/- 0.51 (range, -0.61-1.16). Journals with dedicated profiles and journals with no Twitter presence displayed no significant difference in Impact Factor change between 2015 and 2016 (mean 0.22 vs. 0.15, p=0.58).

Association between Impact Factor, Klout Score, and Alternative metrics

The top-ranked journal by 2016 Impact Factor and the top journal by Twitter Klout Score was The American Journal of Sports Medicine. The number one journal by Altmetric Score was Spine (Table 1).

**Table 1:** Table showing Impact Factor, Twitter Klout Score, Altmetric Score and Twitter data for the 22 Trauma and Orthopaedic journals analysed as part of the study.

Journal Name	Impact Factor 2016	Impact Factor Joining Twitter	Twitter Klout Score	Altmetric Score	Average Number of Tweets Per Day	Total Number of Tweets Retweeted	Total Number of Followers
American Journal of Sports Medicine	5.673	4.439	57	1	0.32	100	18412
Journal of Bone and Joint Surgery - American Volume	4.84	3.427	55	10511	1.56	408	21825
Arthroscopy - The Journal of Arthroscopic and Related Surgery	4.292	3.103	53	10428	0.73	235	7020
Clinical Orthopaedics and Related Research	3.897	2.533	50	15484	1.64	471	5614
Knee Surgery Sports Traumatology Arthroscopy	3.227	2.676	41	6529	0.05	9	527
Bone & Joint Journal	2.948	1.961	52	13889	2.43	695	16920
Journal of Orthopaedic & Sports Physical Therapy	2.825	2.482	53	25798	0.8	251	32251
Physical Therapy	2.764	2.082	49	5843	0.25	72	37643
Journal of Shoulder and Elbow Surgery	2.73	2.319	39	5749	0.27	55	447
Bone & Joint Research	2.597	1.64	47	3909	1.93	238	7543
European Spine Journal	2.563	2.132	41	8369	0.9	76	360
Spine	2.499	2.078	35	28763	2.31	240	2015
Journal of Orthopaedic Trauma	2.251	2.135	37	9332	1.79	455	4296
Journal of Hand Surgery - European Volume	2.191	1.868	47	2132	1.27	125	1588
Clinical Journal of Sports Medicine	2.189	2.11	55	18865	6.01	910	13868
Journal of Spinal Disorders & Techniques	2.042	1.503	25	3798	1.39	55	55
Clinical Biomechanics	1.874	1.874	27	6215	0.01	3	333
Journal of Paediatric Orthopaedics	1.695	1.156	30	5882	1.13	37	105
Journal of Hand Surgery - American Volume	1.606	1.667	44	4406	0.61	117	963
Journal of Foot and Ankle Research	1.405	1.481	44	3988	0.2	52	902
Physician and Sports Medicine	1.292	0.2	42	3670	0.55	125	18559
Orthopedics	1.143	0.962	48	3162	2.59	500	5058

It was found that journals with higher Twitter Klout Scores had higher Impact Factors (R2 0.357, P=0.016). Journals with higher numbers of followers had higher Twitter Klout Scores (R2 0.339, P=0.061). Of the journals with dedicated Twitter profiles, the mean number of Twitter followers was 8923 +/- 2342 (range, 55-37643). The number of followers a journal had did not influence its Impact Factor (R2 0.339, P=0.061). The number of years a journal had been active on Twitter had no association with Twitter Klout Score. The mean number of tweets per day was 1.31 +/- 0.28 (range, 0.01-6.01). The number of tweets per day that a journal produced on Twitter had no correlation with its Impact Factor or with its Twitter Klout Score. The mean number of tweets retweeted was 237.7 +/- 51.4 (range, 3-910). The number of tweets retweeted had no influence on Impact Factor but was associated with having a higher Klout Score (R2 0.509, P=0.008).

The mean Altmetric Score for journals with a dedicated Twitter profile was 8942, compared to 1791 for journals with no Twitter presence (P= <0.001). The mean Altmetric Score for journals with dedicated versus affiliated society Twitter profiles was 8942 and 3711, respectively (p=0.011). No significant difference was seen in Altmetric Scores between journals with affiliated Twitter profiles compared to those with no Twitter presence. A statistically significant correlation was found between journal Altmetric Scores and Impact Factors in 2016 (R2 0.310, P=0.015), and in 2015 (R2 0.334, P=0.009). Having a higher Klout Score was not associated with a higher Altmetric Score, nor was having a higher number of Twitter followers. Journals with higher numbers of tweets were found to have higher Altmetric Scores. Having a higher number of retweets, each retweet being a virtual citation in the Twittersphere was also associated with higher Altmetric Scores (R2 0.463, P=0.015).

## Discussion

This study demonstrates a high level of engagement of Trauma and Orthopaedic Surgery journals with SoMe, with Twitter being the most prevalent SoMe platform in use. Of the leading Trauma and Orthopaedic journals, 72% are represented on Twitter by dedicated Twitter profiles or Twitter profiles of affiliated societies, 66% have Facebook profiles, 44% are represented on Linkedin and 18% have YouTube profiles. For this reason, Twitter was chosen as the SoMe platform most appropriate for analysis in this study. Twitter has been shown to be used not only for professional purposes by board-certified Orthopaedic surgeons and residents but also by members of the public as a means of interaction with their physician and Orthopaedic Surgery as a specialty [13].

Since its development in the 1960’s, Impact Factor has been used as a quantitative measure of journal quality. Essentially, how important a journal is to its end users is equaled to the number and frequency of citations it receives in the scientific literature [14]. However, it is not without limitations [15]. When the end users are researchers publishing papers and articles, this may be an accurate reading of journal appraisal. A journal’s influence on clinical practice relies on its importance to practicing physicians, many of whom do not produce published work as part of a dedicated research agenda [14]. Thus, it is questionable how reliable Impact Factor is a sole measure of journal influence, due to the fact that a key demographic of influence is not taken into account [14-15]. The increase in the use of SoMe by medical professionals provides a platform where this influence can be more broadly assessed.

Given the limited amount of time physicians spend reading scientific journals, SoMe platforms like Twitter help to distill vast quantities of the literature down to key articles and content to view [11]. In addition, dissemination of articles and journal content through Twitter and other SoMe platforms has also been shown to generate increased journal readership [16]. Many scoring systems have been developed since the advent of SoMe as a means of measuring influence and reach across the world-wide-web. One such scoring system is an individual’s Klout Score, as previously mentioned. Systems designed to combine traditional citation metrics and online metrics, such as Altmetrics, have also been developed. As well as quantifying an individual’s reach online and across SoMe, these scoring systems also help to create a broader view of how and to what degree published literature is interacted with online.

Our study found that journals with dedicated Twitter profiles have higher Impact Factors. This is similar to what was shown in a recent study by Kelly, et al. that analyzed Twitter usage among Radiology journals [1]. It may be that journals with higher Impact Factors are more likely to have a Twitter presence, rather than Twitter being wholly responsible for the increase in Impact Factor. Nonetheless, we found that having a dedicated Twitter profile was shown to have a greater effect on a journal’s Impact Factor compared to journals represented by an affiliated society on Twitter. We believe this is because these journals have concentrated Twitter output in relation to their content rather than promotion through a proportion of an affiliated society’s Twitter output.

When an individual produces content on SoMe by way of a post, Tweet, or comment, other users may see this content and perform actions in response to it. The fact that they were prompted to do so implies that the original post influenced them in some manner. Klout Score was developed around this hypothesis [12, 17]. The more Twitter activity and reactions stimulated by a journal’s output on Twitter, the higher its Twitter Klout Score and the greater its degree of influence [12]. Our study found that journals with higher Twitter Klout Scores have higher Impact Factors. The number of followers a journal had was positively associated with its Twitter Klout Score. Similarly, so was the number of tweets a journal produced on Twitter. Looking at numbers of followers and numbers of tweets independently, however, we found that they do not have a direct association with Impact Factor. Rather, they have an indirect influence via Twitter Klout Scores. It may be that highly cited journals are likely to have more followers, tweet more often and, as a result, have more tweets retweeted; a virtual citation. Nonetheless, these parameters were found to positively influence Twitter Klout Scores, thus indirectly influencing journal Impact Factors.

The Klout scoring system is a complicated algorithm that is not published and cannot be reproduced according to its creators [17]. It is limited due to the fact that it amalgamates data from Facebook, Linkedin, and Twitter only. It is widely known that the internet provides a vast array of means of interaction, which a Klout Score does not take into account. Therefore, Klout Scores may not be wholly representative of online influence and have therefore not been adopted as a universal measure.

Altmetric Scores amalgamate web-based data to evaluate the impact of scholarly material online via alternative metrics, such as mentions, clicks, views on social media, Wikipedia citations, downloads, and many other parameters of digital interaction. Our study found that Altmetric Score is positively associated with Impact Factor regardless of whether a journal has a dedicated Twitter profile, affiliated profile or no Twitter presence at all. Journals with dedicated Twitter profiles have significantly higher Altmetric Scores overall. Furthermore, Twitter Klout Score had no correlation with Altmetric Score. This demonstrates that the influence of Altmetric Score on Impact Factor goes deeper than SoMe use alone. Altmetric Scores were found to be high irrespective of the number of followers or Tweets a journal produced. We noted that it is the appeal and relevance of a journal’s online content that drives up its online reach, as it increases the likelihood of interaction, sharing, and dissemination. Unlike Twitter Klout Score, the number of Tweets and followers had no association with higher Altmetric Scores.

Butler, et al. proposed that alternative metrics are likely to play a significant role in literature evaluation going forward alongside traditional metrics [9]. However, the use of alternative metrics is not without limitations [9]. Whilst the number of times an article is cited across the internet is quantified, whether it is a simple mention or an in-depth discussion about the topic is not taken into account. The ‘weight’ of each mention is not accounted for and, therefore, they may vary in relevance. Interest profiles may differ between the articles that are highly interacted with online, compared to those cited by the scientific community, as demonstrated by O’Connor, et al. [18]. The influence of a paper, determined by when it was published, and how topical the subject matter is at the time of mention, is not identified [9, 19]. Furthermore, the validity of Altmetric Score is hindered due to the fact that online data is much easier to manipulate due to the anonymity of the web and lack of quality control across SoMe sites [20]. It is important to ascertain the demographics of those interacting with online research material (the ‘who’), as well as the nature of each interaction (the ‘why’), rather than simply quantifying the number of interactions.

Undoubtedly SoMe provides many positive opportunities for engagement and interaction with medical literature and facilitates its wider dissemination. However, whilst journal content is subject to strict peer review prior to publication, no such rules or constraints exist on SoMe. At the click of a button, information can be posted online for public consumption, whether it is based in fact or not, creating a forum that is open to bias, ego, and abuse. For this reason, users need to act in a mature and responsible manner when interacting with SoMe content. Guidelines have been developed to advise medical professionals on appropriate behavior online [21].

We acknowledge that a limiting factor of this study is the small sample size analyzed. We looked at the Twitter profiles of Trauma and Orthopaedic Surgery journals alone. A broader exploration of these trends across different specialties, and perhaps across more than one SoMe platform, is warranted. We also identified a variation in the levels of journal Twitter activity; some journals produced significantly more output than others on the SoMe site. Furthermore, looking at Twitter and SoMe activity alone is unlikely to give a true representation of the interaction with a journal online, as not everyone uses SoMe [9, 22]. This bolsters the case for the burgeoning strength of alternative metrics in evaluating online interactions with published literature.

## Conclusions

In this study, the use of Twitter and SoMe platforms by leading Trauma and Orthopaedic Surgery journals was investigated. The activity on Twitter and the prevalence of its use among these journals was of key interest. It was found that a positive correlation exists between Impact Factor and the use of Twitter by Trauma and Orthopaedic Surgery journals. It is evident that the use of Twitter and other SoMe platforms provides a means of rapid dissemination of the scientific information, publications, and real-time updates. It also serves to promote international and cross-border engagement, collaboration and interaction around seminal Trauma and Orthopaedic Surgery topics. The engagement with Twitter by Trauma and Orthopaedic Surgeons, and indeed other medical professionals, is to be encouraged as a means of keeping up to date with current literature.

## Figures and Tables

**Figure 1:**
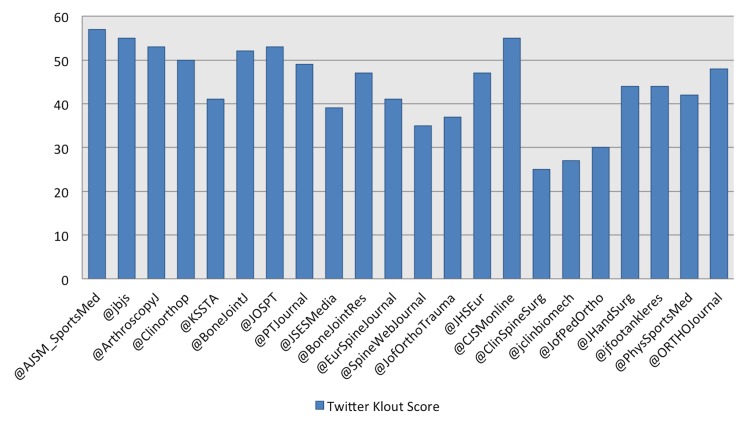
Statistical representation showing Twitter Klout Score by decreasing 2016 Impact Factor.

**Figure 2:**
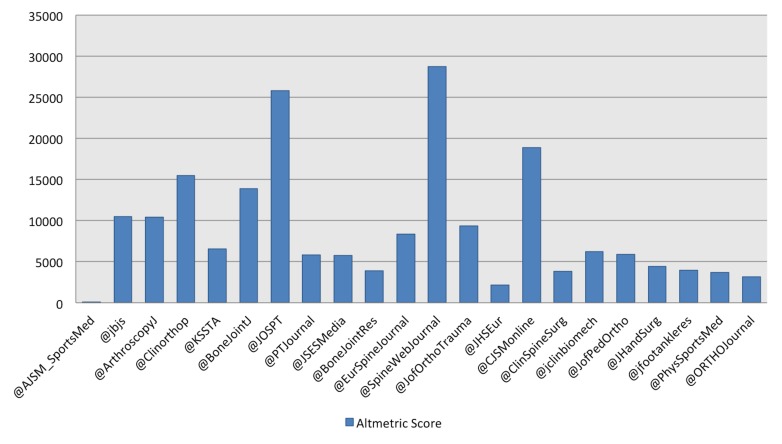
Statistical representation showing Altmetric Score by decreasing 2016 Impact Factor.
