# Correction: The Use of Twitter by Trauma and Orthopaedic Surgery Journals: Twitter Activity, Impact Factor, and Alternative Metrics

**DOI:** 10.7759/cureus.c13

**Published:** 2018-06-15

**Authors:** Hannah Hughes, Andrew Hughes, Colin G Murphy

**Affiliations:** 1 Department of Trauma and Orthopaedic Surgery, St. Vincent's University Hospital, Dublin, Ireland; 2 Department of Trauma and Orthopaedic Surgery, Galway University Hospital; 3 Department of Trauma and Orthopaedic Surgery, Galway University Hospital , Galway, IRL

Due to severe editor error, the following grammatical typos and inconsistencies were introduced during copy editing and have now been fixed in the udpated version of the article:

- Multiple instances of the word "the" introduced when grammatically incorrect
- Each mention of 'Klout Score' and 'Altmetric Score' should have had both words capitalized.

The following errors were not corrected during copy editing, but have now been addressed in the updated version of the article:

- Abstract, Results section, first sentence: "were dedicated to the Twitter profiles" changed to 'had dedicated Twitter profiles."
- Introduction, first paragraph, first sentence: "are" changed to "is"
- Introduction, first paragraph, second sentence, "nearly" changed to "near"
- Introduction, third paragraph, second sentence: comma added after "Impact Factor"
- Materials and Methods, first paragraph, first sentence: "in the" changed to "of"
- Materials and Methods, first paragraph, fourth sentence: "twitter" has been capitalized
- Materials and Methods, first paragraph, fourth sentence: "were recorded." has been deleted
- Materials and Methods, second paragraph, fourth sentence: comma added after "limited to"
- Materials and Methods, fifth paragraph, fifth sentence: commas deleted after "time" and "mentions"
- Discussion, first paragraph, third sentence: "SoMe most appropriate platform" changed to "SoMe platform most appropriate to analysis"
- Discussion, second paragraph, second sentence: "by" changed to "to"
- Discussion, third paragraph, first sentence: comma deleted after "time"
- Discussion, eighth paragraph, fourth sentence: "does not account for" changed to "is not accounted for"
- Discussion, ninth paragraph, first sentence: "provide" changed to "provides"
- Discussion, ninth paragraph, third sentence: "it's" changed to "it is"
- Discussion, tenth paragraph, second sentence: comma added after "platform"
- Conclusions, first paragraph, last sentence: comma added after "professionals"

Cureus sincerely regrets these errors and has taken steps to ensure this will not happen again. Copies of the original, unaltered article are available upon request. Please contact for a copy of the unaltered article.

